# Lived Experiences Versus Artificial Intelligence to Reduce Hospital Readmission: A Qualitative Study

**DOI:** 10.1093/geroni/igaf122.1486

**Published:** 2025-12-31

**Authors:** Elham Mahmoudi

**Affiliations:** University of Michigan, Ann Arbor, Michigan, United States

## Abstract

Frequent hospitalization including hospital readmissions are expensive and harmful to older adults, particularly those with dementia. Despite efforts to predict and reduce hospital readmissions through novel applications of machine learning (ML), readmission remains elusive. This study stemmed from our prior unsuccessful work and work of others attempting to use ML to predict and, by acting upon that, reduce hospital readmission among people with dementia. To understand what was missing in our ML models, we used a qualitative descriptive study and interviewed 21 participants (n = 11 caregivers of people with dementia; n = 10 hospital staff including nurses, discharge planners, and social workers). Participants took part in a semi-structured interview. To analyze the data, we used thematic text analysis to identify overarching themes. We identified five themes related to high risk of readmission among patients with dementia: (1) caregivers’ burdens of providing dementia care, (2) lack of or inadequate support for caregivers, (3) lack of evidence-based educational materials for caregivers, (4) inadequate dementia-care protocols and guidelines across all healthcare settings, and (5) shortage of trained work force for dementia care. Mainly, in the absence of accessible, affordable, and dementia-specific outpatient medical care and respite centers, emergency departments and hospitals are caregivers only choices when care at home becomes too challenging. Recommendations provided by the caregivers and hospital staff to reduce readmission for patients with dementia include: (1) specialized wards in hospitals for dementia patients; (2) dementia-specific inpatient/discharge protocol with educational materials for caregivers, (3) at-home visits/follow-up, and (4) accessible and affordable home care services.

